# Application of arthroscopic system in the treatment of lactational breast abscess

**DOI:** 10.1186/s12893-022-01845-z

**Published:** 2022-11-18

**Authors:** Longquan Lou, Wei Ma, Xiaojin Liu, Haibin Shen, Haiming Wang, Hao Lv

**Affiliations:** 1 Department of General Surgery, Hangzhou Third People’s Hospital, 38 West Lake Avenue, Hangzhou, 310000 Zhejiang China; 2 Department of Osteology, Hangzhou Third People’s Hospital, 38 West Lake Avenue, Hangzhou, 310000 Zhejiang China

**Keywords:** Lactational breast abscess, Arthroscopic system, Minimally invasive therapy

## Abstract

**Purpose:**

Optimal treatment of breast abscesses has been controversial. Herein, we report an innovative method for the operative treatment of lactational mammary abscesses.

**Methods:**

Nineteen lactating patients diagnosed with breast abscesses were enrolled in the study, and abscess debridement and drainage were performed using an arthroscopic system. The clinical characteristics of the patients were recorded to evaluate the feasibility, efficacy, and cosmetic results of arthroscopic surgery for breast abscesses.

**Results:**

All 19 patients were cured and did not relapse within the 6-month-follow-up period. One patient stopped breastfeeding due to breast leakage. All patients were satisfied with the postoperative appearance of the breast.

**Conclusion:**

Arthroscopic debridement and drainage are effective treatment methods for lactational breast abscesses, with a high cure rate, few complications, and satisfactory cosmetic outcomes.

## Introduction

Most cases of acute mastitis occur in lactating women, and approximately 0.4–11% of patients eventually develop breast abscesses [1, 2]. Surgical incision and drainage (I&D) was once the recommended management for mammary abscesses [3]. However, it has been found to be associated with interruption of breastfeeding, formation of breast fistula, prolonged healing time, and obvious scarring [4, 5]. Recently, clinicians have reported many minimally invasive treatment methods, such as fine-needle aspiration [6], percutaneous catheter placement [7, 8], and vacuum-assisted breast biopsy (VABB), for breast abscesses [9, 10]. However, these minimally invasive treatments often fail, especially in cases of large (> 3 cm in diameter) or multilocular mammary abscesses [11, 12]. Therefore, a treatment method that can ensure adequate drainage and result in satisfactory cosmetic outcomes needs to be developed.

Arthroscopy is a minimally invasive procedure that enables the surgeon to see the joint through a 5-mm incision [13]. In addition, the surgeon can use this method to insert specialized surgical instruments through additional incisions to repair some types of joint diseases [14, 15]. Currently, arthroscopy has been widely used for the diagnosis and treatment of various intra-articular diseases, such as knee joints, wrist joints, and finger joints [16].

In this study, we aimed to investigate the feasibility, safety, efficacy, and aesthetics of arthroscopic surgery for the treatment of lactational breast abscesses.

## Patients and methods

### Patients

Between January 2019 and January 2021, 19 consecutive female patients diagnosed with lactational breast abscess at Hangzhou Third People’s Hospital were recruited for this prospective, nonrandomized study. Lactational breast abscess is a female-specific disease. Thus, no men were involved in the study. This study was approved by the Ethical Committee of Hangzhou Third People’s Hospital, and informed consent was obtained from all patients.

Patient eligibility criteria included the following: (1) lactating women with breast pain or malaise; (2) manifestation of clinical symptoms including redness, warmth, tenderness, and induration; (3) presence of a fluctuant mass; and (4) preoperative ultrasonographic examination findings of an abscess cavity with a diameter ≥ 3 cm.

### Surgical instruments

The surgical instruments included an ultrasound machine (Mindray, China), an arthroscopic system (Smith&Nephew, USA), an endoscopic radiofrequency ablation system (Smith&Nephew, USA), and an arthroscopic shaver system (Smith&Nephew, USA).

### Surgical procedures

**Fig. 1:**
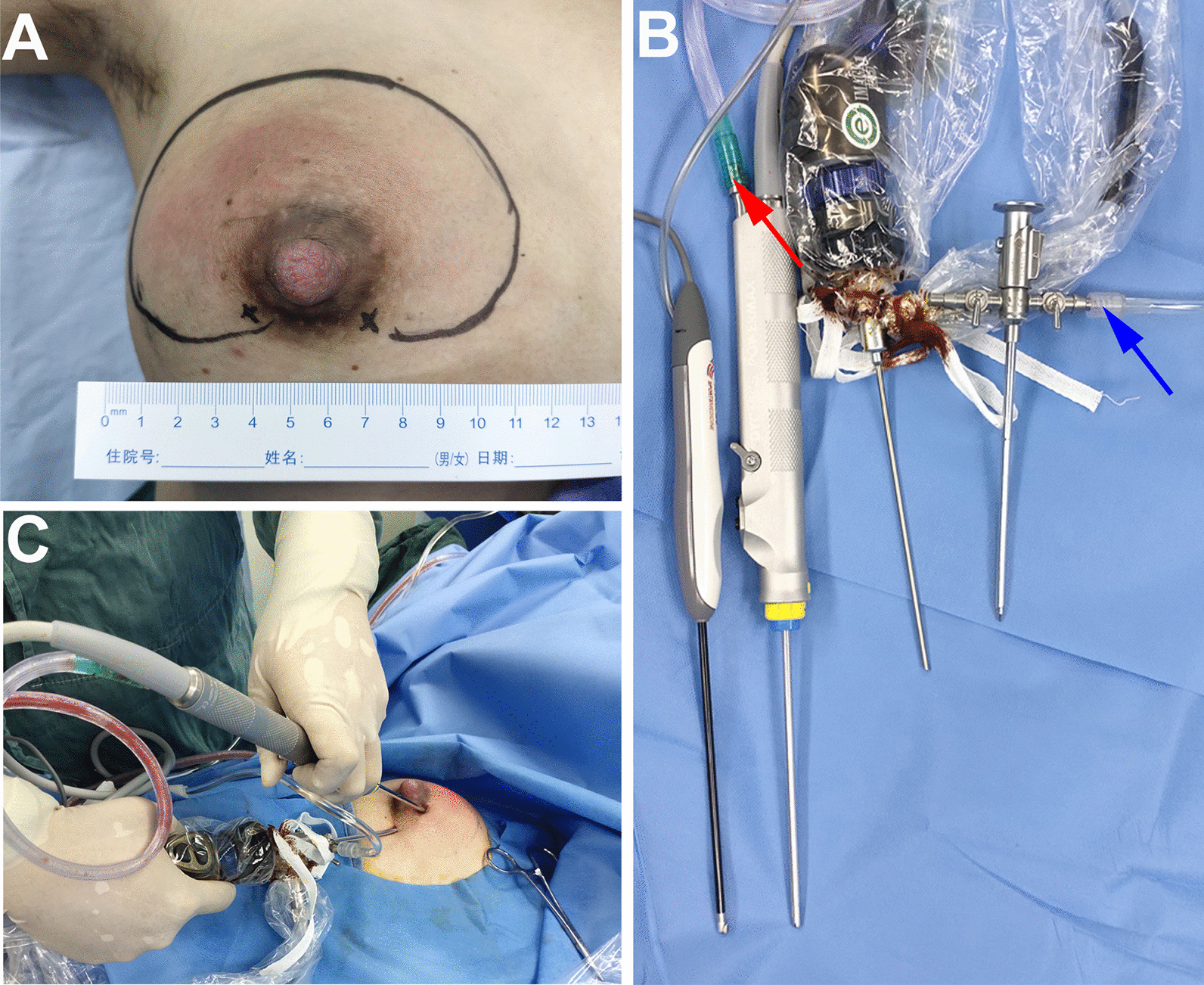
Surgical preparation and operation scheme. **A** The surface location of the abscess was marked under the guidance of an ultrasound; **B** Instruments required for the operation (from left to right): Endoscopy radio frequency ablation wand, Arthroscopy shaver (red arrow: connected to the negative pressure aspirator), Arthroscopy, and Sheath (blue arrow: connected to the saline perfusion pump); C: Schematic diagram of the surgical procedure

**Fig. 2:**
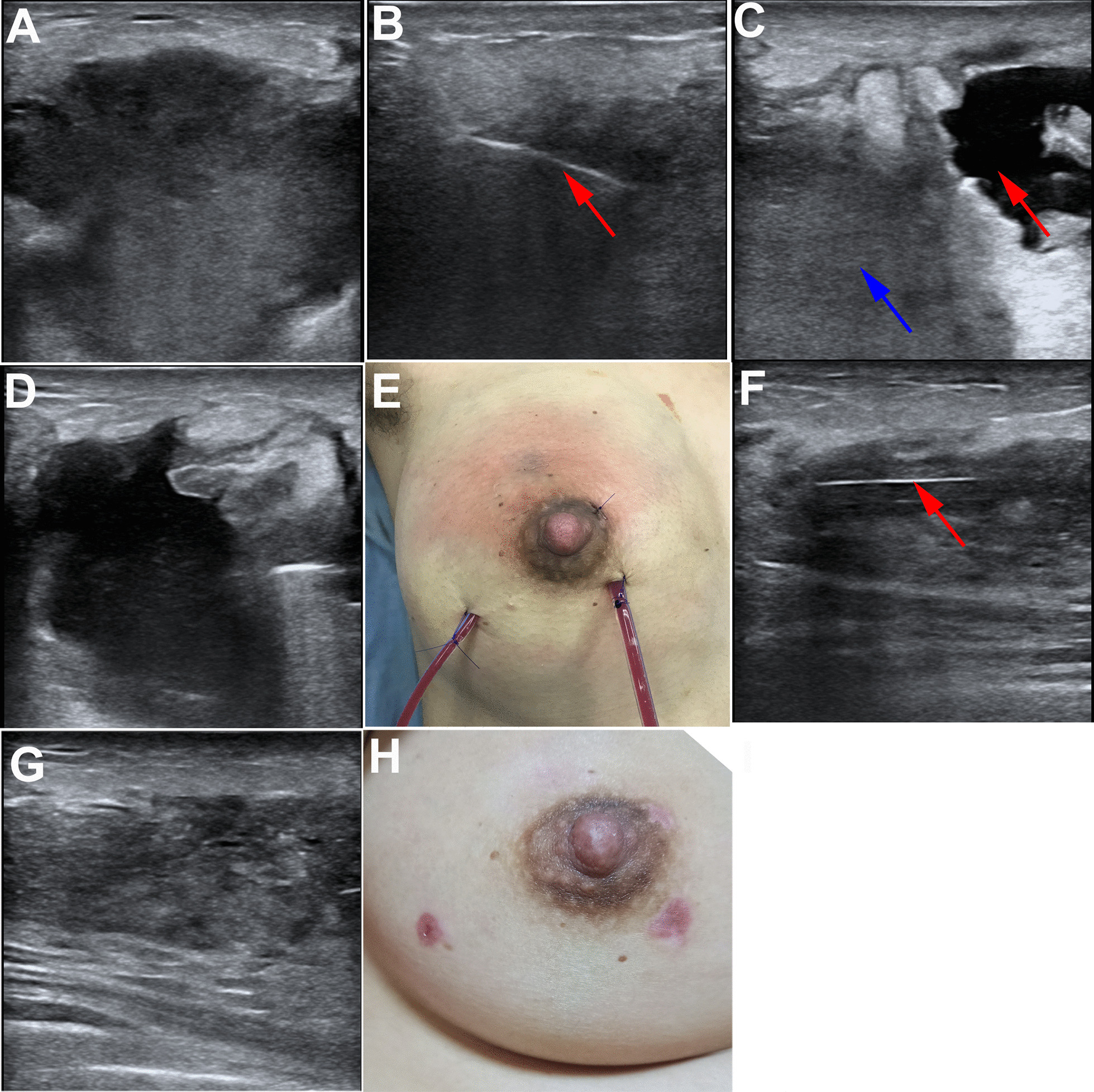
Ultrasound-assisted throughout the management. **A** Preoperative ultrasonography was used to locate the abscess cavity; **B** Ultrasound-guided placement of the arthroscopic sheath (red arrow); **C** The abscess cavity (red arrow) and its adjacent abscess cavity (blue arrow) was surgically cleared (red arrow); **D** The septum between the abscess cavities was opened under ultrasound guidance; **E** One or two drainage tubes was placed for continuous drainage; **F** Ultrasound examination on the first day after surgery showed the drainage tube in the original abscess cavity (red arrow); **G** Ultrasonography was performed after the drainage tube was removed. **H** Breast appearance at 2 weeks postoperatively

**Fig. 3:**
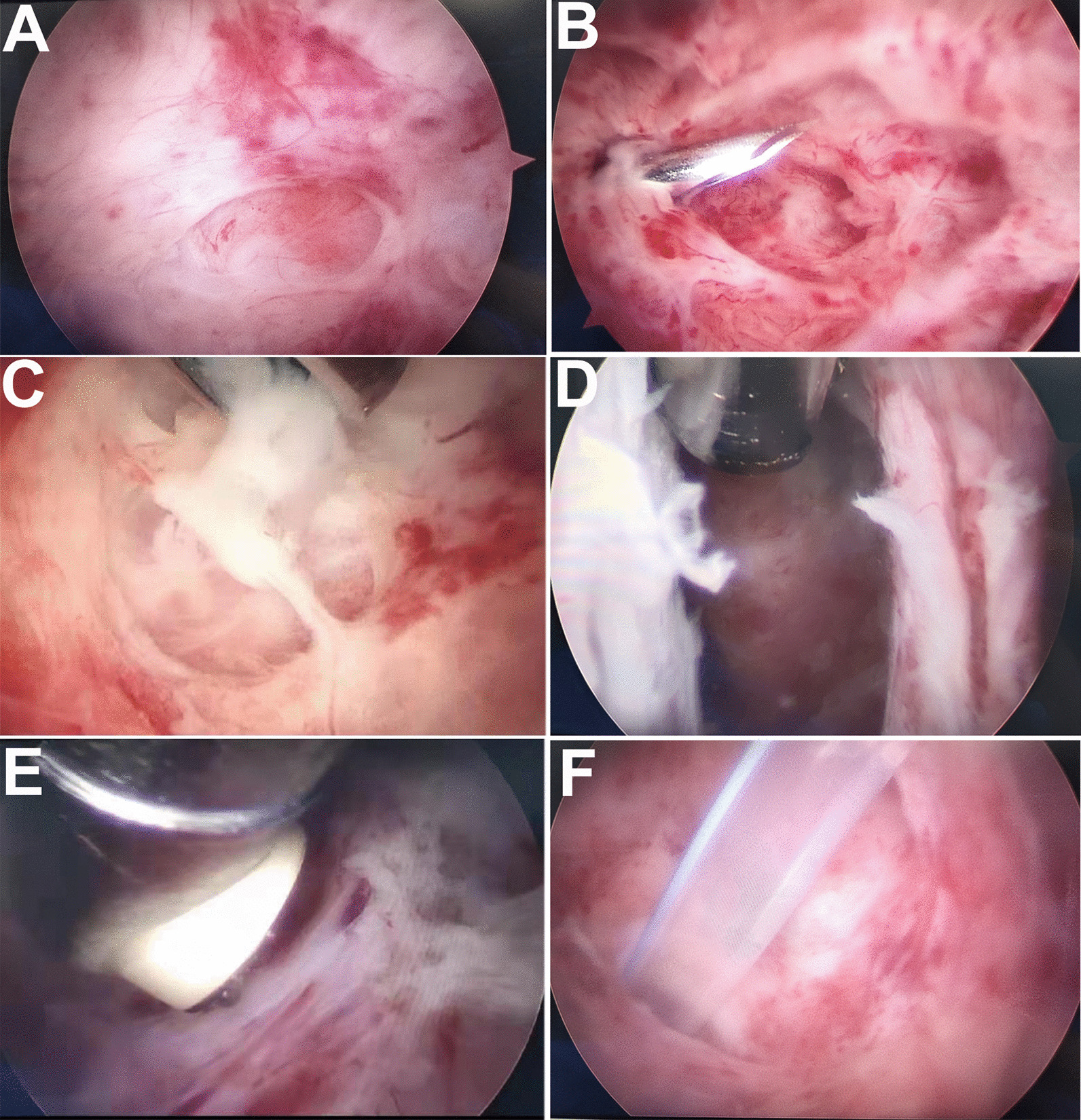
Endoscopic cleansing of the abscess cavity. **A** The endoscopic view of the abscess cavity after discharge of pus; **B** Specialized surgical instruments were inserted under endoscopic vision; **C** The necrotic tissue was drawn in with negative pressure and removed by the rotating blade of the arthroscopy shaver; **D** The septum between the abscess cavities was opened; **E** The radiofrequency ablation probe was used for hemostasis; **F** The drainage tube was placed appropriately

All operations were performed jointly by a general surgeon and an orthopedic surgeon. Patients were placed in the supine position with the upper limb/surgical side in abduction. Before surgery, the extent of the abscess was marked under ultrasound guidance. An incision was subsequently made (Fig. 1A). The surgical instruments (Fig. 1B) and surgical schematic (Fig. 1C) are shown in Fig. 1. Periareolar incisions were made to treat the abscesses in the upper part of the breast whereas inframammary incisions were used to treat abscesses in the lower portion of the breast. After sterilization, a mixture (< 100 mL) of 1% lidocaine hydrochloride and epinephrine (1:200,000) was injected around the abscess for local anesthesia. After skin incision, an arthroscopic sheath, which was connected to a saline perfusion pump with a pressure of 30–40 mmHg, and an arthroscopic shaver were implanted into the abscess cavity under ultrasound guidance (Fig. 2A, B). First, the pus was drained using an arthroscopic shaver, which was connected to the negative pressure aspirator, until the clarified saline was extracted. This allowed the surgeon to carefully explore the interior of the abscess cavity by arthroscopy (Fig. 2C. 3A). If necessary, additional manipulative instruments (Fig. 1B) can be inserted under the guidance of the arthroscope (Fig. 3B) to handle more complex situations, such as multilocular abscesses. Second, an arthroscopic shaver was used to clean the necrotic tissue on the abscess wall (Fig. 3C) and open the septum between the abscess cavities (Figs. 2D and 3D). During this process, endoscopic radiofrequency ablation was used to eliminate bleeding points (Fig. 3E) and breakage of the milk ducts. Finally, one or two drainage tubes were placed through the original incision or additional incision to achieve adequate drainage (Figs. 2E and 3F).

Catheters were removed when the drainage volume was < 10 mL/day for two consecutive days, and no residual abscess was confirmed via ultrasound (Fig. 2F, G).

### Supportive measures

All patients received immediate empirical oral antibiotics. In addition, sensitive antibiotics were administered based on bacterial culture results. Patients were encouraged to start breastfeeding 24 h after the surgery to prevent milk siltation and were instructed to keep the nipple clean to prevent bacterial invasion.

### Follow-up

Patients were followed up via outpatient or telephone appointments for at least six months. The 1–3-day pain score (0–10) after surgery, duration of drainage, time of start of breastfeeding, and complications were recorded. All patients were required to grade their satisfaction with the postoperative appearance. The grading was as follows: poor (0), average (1), good (2), or excellent (3).

## Results

### Clinical characteristics

In this study, we performed arthroscopic surgery in 19 patients with mammary abscesses. The clinical characteristics of the patients are shown in Table 1. The mean age of the included patients was 29.6 ± 5.2 years in age (range 22–42). Twelve (63.2%) patients were primiparous, whereas seven (36.8%) were multiparous. The average diameters of 11 unilocular abscesses were 44.3 ± 9.2 mm. The sum of the lumen diameter of the nine multilocular abscess cases was 81.1 ± 28.7 mm.

**Table 1:** Clinical characteristics of included patients

Characteristics	N = 19				Mean ± SD [range]
Age (years)	29.6 ± 5.2 [22–42]				
Parity	Primiparae	Multiparae			
	12 (63.2%)	7 (36.8%)			
Abscess cavity	Single	Multiple			
	11(57.9%)	8 (42.1%)			
Diameter of abscess (mm)	44.3 ± 9.2 [36–65]	81.1 ± 28.7 [41–120]			
Pain score (0–10)	Day1	Day2	Day3		
	2.10 ± 1.02 [1–5]	1.15 ± 0.93 [0–4]	0.52 ± 0.35 [0–3]		
Duration of drainage (days)	4.52 ± 1.23 [3–7]				
Breastfeeding	Within 1 week	Within 2 weeks	Stop		
	13 (68.4%)	18 (94.7%)	1 (5.3%)		
Complications	Breast leakage	Hemorrhaging	Relapse	Distortions	None
	1 (5.3%)	0	0	0	18 (94.7%)
Satisfaction	Poor	Average	Good	Excellent	Score
	0	0	2 (10.5%)	17 (89.5%)	2.9 ± 0.3

### Therapeutic effect

All patients were cured and did not relapse within the 6-month follow-up period. The pain scores at days 1, 2, and 3 after surgery were 2.10 ± 1.02, 1.15 ± 0.93, and 0.52 ± 0.35, respectively (Table 1). The average drainage time was 4.52 ± 1.23 days (Table 1). The daily discharge range was 2–45 mL, and the total drainage range was 28–165 mL. One patient developed breast leakage, which was defined as the presence of milk in the drainage fluid. The drainage tubes were removed after the breast leakage disappeared upon the administration of bromocriptine. The drainage fluid of the remaining 18 patients was uniformly reddish or yellowish with a small amount of necrotic tissue. Except for the patient who stopped breastfeeding due to milk leakage, the remaining 18 patients resumed breastfeeding within 2 weeks, while 13 of them resumed breastfeeding within 1 week. There were no complications, such as hemorrhaging and breast distortion (Fig. 2H). All patients were satisfied with the postoperative breast appearance, and the satisfaction score was 2.9 ± 0.3.

## Discussion

Breast abscess is an acute suppurative inflammation that mainly develops from lactational mastitis and usually requires surgical treatment [1, 17]. Traditional treatment requires extensive I&D, which often results in severe pain, breast deformation, and lactation interruption [5, 18]. Therefore, minimally invasive treatment of breast abscess is constantly being explored in the clinic [4]. At present, the reported minimally invasive treatment modalities of breast abscess include needle aspiration [19], percutaneous catheter placement [7, 8], and VABB surgery [9, 17].

Needle aspiration is recommended for the treatment of univentricular abscesses with a diameter of < 3 cm [4]. If the abscess size is > 5 cm, the failure rate of needle aspiration therapy is 50–75% [11, 12]. Surgical I&D should be considered as first-line therapy in treatment of large (> 5 cm) or multilocular abscesses [4]. Studies have shown that catheter placement has a high cure rate of 85–88.2% for unilocular abscesses ≥ 3 cm [20–22], indicating that continuous drainage is the key treatment for abscesses. However, in multilocular abscesses, aspiration and catheter placement are ineffective [11]. Although VABB can be used to treat multilocular abscesses, it tends to damage the milk ducts and normal breast tissue, thereby leading to breast leakage [21]. In addition, micro-incision drainage, as reported by Liu Jun et al., has a higher failure rate in the treatment of breast abscesses > 5 cm [19]. Therefore, traditional surgical drainage is an inevitable procedure in patients with multilocular abscess or in those in whom minimally invasive treatment failed [4].

The arthroscopic system is an endoscopic system for the treatment of intra-articular diseases [13]. The arthroscope can enable the surgeon to see the inside of the joint cavity through a small incision. Moreover, it is connected to a saline pump to continuously rinse the joint cavity to maintain a clear field of vision [23]. If necessary, a variety of supporting instruments can be used to clean the interior of the joint cavity and to repair joint injury [15, 24]. In this study, an arthroscopic system was applied for the treatment of breast abscesses for the first time, and satisfactory results were obtained. Our study showed that arthroscopy is suitable for unilocular abscesses with a diameter > 3 cm and for complex multilocular abscesses. All patients were cured after arthroscopic treatment. This may be attributed to the thorough removal of necrotic tissue in the abscess cavity, continuous irrigation with normal saline during the operation, and continuous drainage after surgery. Because the normal breast tissue is minimally damaged, the pain after arthroscopy is tolerable for patients. Usually, patients are encouraged to resume breastfeeding as early as possible and to clean their nipples regularly. There were no hemorrhaging, recurrence, nor breast deformation in any of the patients. In fact, all of the patients were satisfied with the postoperative appearance of their breasts.

To avoid treatment failure, we usually removed the drainage tube if the drainage volume was less than 10 mL/d for two consecutive days, and if the ultrasound findings confirmed that there was no residual abscess. Our study showed that continuous drainage was required for 4.52 ± 1.23 [3–7] days after arthroscopic surgery [10]. Similar to our study, catheter drainage was required for 4.4 ± 1.3 [3–8] days after the Encor procedure, which was a type of VABB system. In contrast, the Mammotome procedure, which is another VABB system, usually requires postoperative catheter drainage for 14 days [9]. The duration of drainage required for catheter drainage without surgery has been reported inconsistently, ranging from 1 to 25 days [21, 25]. These results could not be directly compared due to the differences in the drain removal criteria. However, in general, arthroscopic surgery significantly reduced the maximum duration of catheter placement.

Breast leakage is a common complication in the surgical treatment of breast abscess [17, 26], defined as the presence of breast milk in the drainage fluid. Breast leakage require prompt management to avoid the development of milk fistula. In our study, one (5.3%) patient with multilocular abscess developed milk leakage, which might be related to the damage of milk ducts during the interval of surgical separation of the abscess. The patient’s breast leakage resolved after the administration of bromocriptine; hence, the drainage tube was removed on postoperative day 7. 28.2% of patients developed milk leakage after ultrasound-guided aspiration or catheter placement [21]. Zhu et al. reported that the incidence of postoperative breast leakage was 42.3% after mammotome surgery and 43.5% after I&D for lactating breast abscesses [17], with no statistical difference. Interestingly, the incidence of breast leakage after the Encor surgery was only 5.6% [10]. The mechanism of such a large difference in the incidence of breast leakage after surgery with different VABB systems remains unclear. The I&D and VABB procedures could not result in the removal of necrotic tissue without damaging the normal tissue. Arthroscopic surgery presents a real-time view of the abscess interior, which avoids blind destruction of the normal tissue. In addition, radiofrequency ablation was used to directly close the lacteal duct injuries found during the procedure to minimize the occurrence of milk leakage.

Furthermore, the surgical influence on further lactation is also a matter of concern. The probability of termination of breastfeeding after I&D has been reported to be as high as 46–100% [27, 28], 34.5–57.9% after ultrasound-guided catheter drainage [21, 29], and 30.6% after VABB surgery[10]. In our study, one (5.3%) patient stopped breastfeeding due to milk leakage, and the remaining 18 (94.7%) patients eventually resumed breastfeeding. Although patients are encouraged to resume breastfeeding as early as possible in this study, they may delay breastfeeding due to pain and discomfort, psychological resistance, and babies refused to suckle. Comparatively, arthroscopic surgery was more favorable for the resumption of postoperative breastfeeding.

In terms of long-term cosmetic outcomes, none of the minimally invasive treatment methods, including percutaneous aspiration, catheter drainage, arthroscopic surgery, and VABB surgery, resulted in significant breast deformation after successful treatment [21]. In contrast, the probability of breast deformation after I&D was 16.7% [19], and as high as 70% [12] of the patients were dissatisfied with the cosmetic results.

Our study showed a cure rate of 100% with no recurrence after arthroscopic surgery. Similarly, the VABB procedure had a cure rate of 94.7–100% and a recurrence rate of 0–3.8% [9, 10, 17, 30]. Both arthroscopic and VABB procedures achieved the therapeutic effects of I&D. However, arthroscopic surgery is less invasive and associated with a shorter postoperative drainage time, which makes it more suitable for the treatment of lactational breast abscesses.

This study suggests that arthroscopic surgery can be used as a potential treatment for unilocular abscesses > 3 cm and for complex multilocular abscesses. However, there are no clear conclusions because this was a consecutive case study, and the number of patients included is small. In the future, a randomized controlled trial involving more patients is needed to identify the advantages of arthroscopic surgery.

In conclusion, arthroscopic debridement and drainage are potential treatments for lactational mammary abscesses with curative effects similar to those of traditional surgery.

## Data Availability

Please contact the corresponding author for data requests.
